# Comparison of plug-based versus suture-based vascular closure for large-bore arterial access: a collaborative meta-analysis of observational and randomized studies

**DOI:** 10.1007/s00392-022-02145-5

**Published:** 2023-02-07

**Authors:** Oliver Dumpies, Alexander Jobs, Danilo Obradovic, Maarten van Wiechen, Philipp Hartung, Johannes Rotta detto Loria, Johannes Wilde, Nicolas Majunke, Philipp Kiefer, Thilo Noack, Holger Thiele, Nicolas van Mieghem, Steffen Desch, Mohamed Abdel-Wahab

**Affiliations:** 1 Department of Cardiology, Heart Center Leipzig at University of Leipzig, Leipzig, Germany; 2 German Centre for Cardiovascular Research (DZHK), Partner Site Hamburg/Luebeck/Kiel, Hamburg, Germany; 3 Department of Cardiology, Erasmus University Medical Center, Rotterdam, Netherlands; 4 Department of Cardiac Surgery, Heart Center Leipzig at University of Leipzig, Leipzig, Germany

**Keywords:** Large-bore vascular closure, MANTA, ProGlide, Vascular closure device, Vascular complications

## Abstract

**Background:**

Large-bore arteriotomies can be percutaneously closed with suture-based or plug-based vascular closure device (VCD) strategies. The efficacy of both techniques remains controversial.

**Aims:**

We conducted a meta-analysis of comparative studies between both VCD strategies, focusing on the most commonly applied VCDs (MANTA and ProGlide).

**Methods:**

We searched MEDLINE, the Cochrane Central Register of Controlled Trials and Google scholar for observational studies (OS) and randomized controlled trials (RCT) comparing vascular closure with the MANTA-based and the ProGlide-based technique. The principal endpoint of this analysis was access-site related vascular complications. Both study types were analyzed separately.

**Results:**

Access-site related vascular complications were less frequent after vascular closure with the MANTA technique in the analysis of OS (RR 0.61 [95%CI 0.43–0.89], *p* = 0.01, *I*^2^ = 0%), but more frequent in the analysis of RCT data (RR 1.70 [95%CI 1.16–2.51], *p* = 0.01, *I*^2^ = 0%). Both data sets provided no significant difference between the VCD techniques in terms of overall bleeding events (OS: RR 0.57 [95%CI 0.32–1.02], *p* = 0.06, *I*^2^ = 70%; and RCT: RR 1.37 [95%CI 0.82–2.28], *p* = 0.23, *I*^2^ = 30%). RCT data showed that endovascular stenting or vascular surgery due to VCD failure occurred more often after MANTA application (RR 3.53 [95%CI 1.07–11.33], *p* = 0.04, *I*^2^ = 0%).

**Conclusions:**

While OS point to favorable outcomes for large-bore vascular closure with the MANTA-based technique, RCT data show that this strategy is associated with more access-site related vascular complications as well as endovascular stenting or vascular surgery due to device failure compared with the ProGlide-based technique.

**Graphical abstract:**

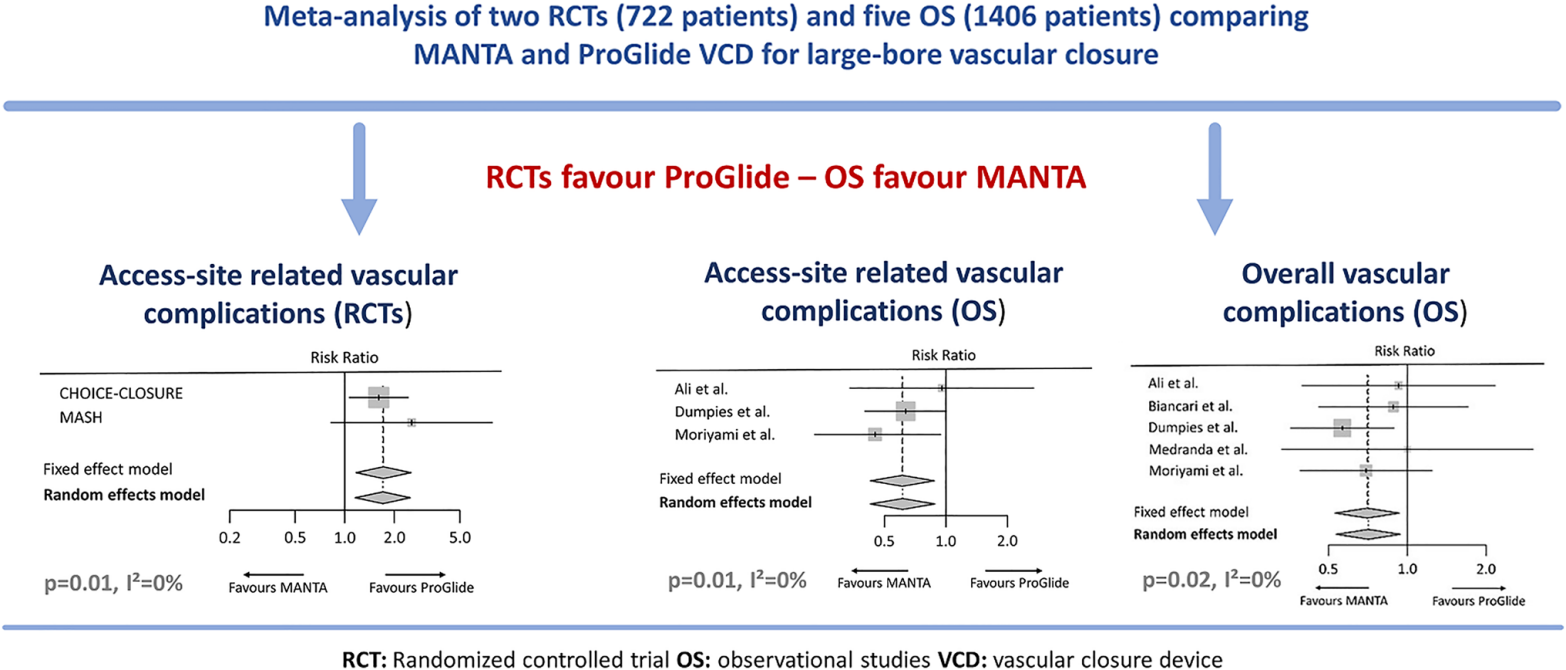

**Supplementary Information:**

The online version contains supplementary material available at 10.1007/s00392-022-02145-5.

## Introduction

Suture-based vascular closure devices (VCDs) have been the sole option for non-surgical percutaneous large-bore arterial access-site closure for several years. The Perclose ProGlide VCD (Abbott Vascular, Chicago, Illinois, USA) is most commonly used, and appears to be superior to other suture-based techniques [1, 2]. It is commonly applied in several percutaneous procedures requiring large-bore vascular access, such as transcatheter aortic valve implantation (TAVI) [1, 2], venoarterial extracorporeal membrane oxygenation (va-ECMO) [3], and endovascular aortic repair (EVAR) [4]. But despite increasing operator experience and improved procedural techniques, vascular complications due to suture-based VCD failure remain common and are associated with significant morbidity and mortality [5, 6], which necessitated the development of alternative VCD technologies.

The MANTA VCD (Teleflex, Wayne, Pennsylvania, USA) is the most studied of the recently introduced newer generation large-bore VCDs. It relies on access-site closure using a collagen plug, an approach that is similar to smaller plug-based VCDs [7]. A growing number of observational studies (OS) reported that the MANTA-based closure technique is associated with comparable or less vascular complications compared to the ProGlide-based technique [6, 8–13]. These studies were followed by the randomized MASH trial, which did not find significant differences in access-site related bleeding and vascular complications between both VCD strategies [14]. The larger randomized CHOICE-CLOSURE study showed that access-site related vascular complications occurred more frequently in patients receiving the MANTA VCD [15].

Consequently, meta-analyses comparing both VCDs have been recently performed [16–18], but none of them found a difference in terms of vascular complications or bleeding events between both techniques. However, these meta-analyses have important methodological limitations, particularly by mixing OS and randomized controlled trials (RCTs). Therefore, we sought to compare the plug-based MANTA technique and the suture-based ProGlide technique based on published data from both OS and RCTs in a collaborative meta-analysis, but with separate analyses of the OS and RCTs.

## Methods

### Search strategy and selection criteria

We searched PubMed, Google Scholar, and the Cochrane Central Register of Controlled Trials for reports published between January 1, 2014, and November 1, 2021. We set the start of the search period two years before the CE certification of the MANTA VCD. Three term groups were used, of which at least one term of each group was required to match: (1) vascular closure; vascular closure device; vascular; large-bore arteriotomy; large bore arteriotomy; large-bore arteriotomies; large bore arteriotomies; percutaneous closure; percutaneous AND (2) MANTA; plug-based; plug based; plug AND (3) ProGlide; Perclose; suture-based; suture based; suture.

We included peer-reviewed RCTs as well as peer-reviewed OS comparing the plug-based MANTA and the suture-based ProGlide vascular closure techniques after large-bore vascular access. Only studies reporting vascular complications according to the Valve Academic Research Consortium (VARC)-2 criteria were included [19]. Studies that did not differentiate between major and minor vascular complications or whose results did not clearly present this differentiation were excluded.

### Data extraction

Two independent investigators (OD, DO) performed the literature search using the previously defined search terms. Studies that did not meet the eligibility criteria were excluded. Discrepancies were resolved by consensus after discussion. Data extraction from available full-text articles was also carried out independently by two investigators (OD and DO) using piloted spreadsheets. Once again, discrepancies were resolved by consensus after discussion. In addition, unpublished data from the CHOICE-CLOSURE trial [15] and the observational study by Dumpies et al. [9] were included if necessary. Two investigators (OD and DO) independently assessed the risk of bias of the studies according to the Cochrane Collaboration's tools for non-randomized studies [20] (supplementary table 1) and randomized studies [21] (supplementary table 2).

### Study outcomes

The principal endpoint of this analysis is access-site related vascular complications defined according to VARC-2. OS only partially differentiated between access-site related and non-access-site related vascular complications. Therefore, we also assessed the occurrence of overall vascular complications in this data set.

Secondary endpoints included all-cause mortality, overall-, life-threatening or major and minor bleeding events (VARC-2), VCD failure (VARC-2), as well as endovascular stenting and vascular surgery due to VCD failure.

As the MASH trial did not provide information on the distribution of life-threatening, major and minor bleeding events, only the rates of overall and access-site related bleeding events were reported in the RCT group. The two RCTs used different definitions of VCD failure. Therefore, we only matched the need for endovascular stenting and vascular surgery due to VCD failure. In addition, mortality data in the MASH trial have not been reported. A detailed list of all outcome definitions, inclusion and exclusion criteria for each study is provided in supplementary tables 3 and 4.

### Data analysis

OS and RCTs were analyzed separately. For the principal and secondary outcomes, risk ratios (RR) were calculated based on the number of events and the number of patients per group. These study-level results were pooled by means of a random effects meta-analysis using the Mantel–Haenszel method as primary analysis. Between-study variance was estimated using the Paule-Mandel estimator. The result of a fixed-effect meta-analysis is reported in addition. In case of evidence for a small-study effect and a more conservative result of the fixed-effect meta-analysis as compared to the random effects meta-analysis, primary interpretation was based on the result of the fixed-effect meta-analysis. Cochran’s *Q* statistic and Higgins and Thompsons *I*^2^ were calculated to assess heterogeneity. We used R (version 4.1.2) and its package meta (version 5.1-1) for all statistical analyses.

## Results

**Fig. 1:**
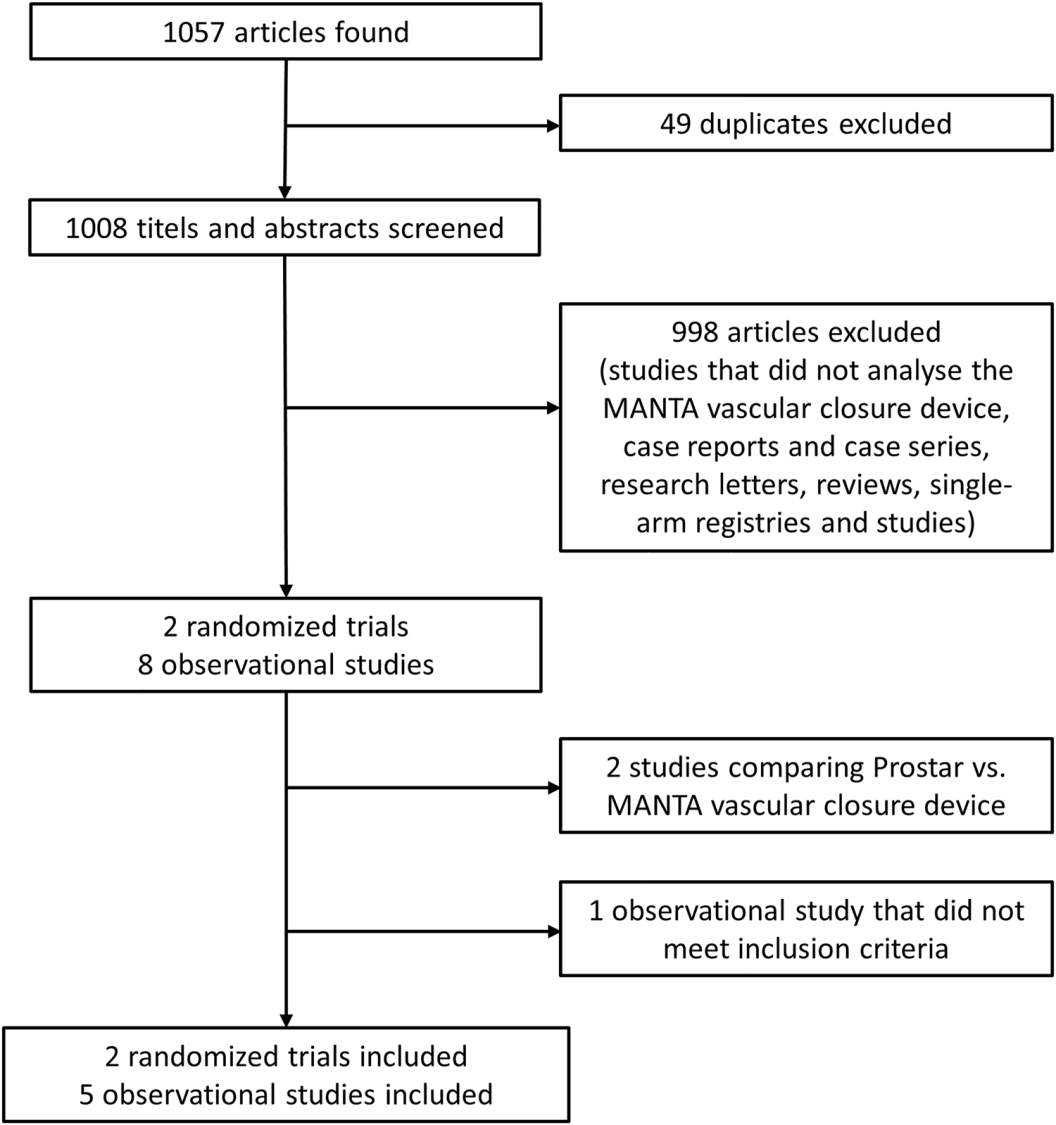
Trial selection

Our search identified 1057 articles, of which two RCTs and eight OS remained after excluding duplicates and studies not meeting the above-mentioned selection criteria. Two OS [22, 23] were excluded because they compared the MANTA VCD with the ProStar VCD. One observational study [12] had to be ruled out, as the data available to us could not provide a clear conclusion about major and minor vascular complications according to the VARC-2 criteria. The study selection process is described in Fig. 1. Ultimately, we included five OS with a total of 1406 patients comparing the MANTA (*n* = 587) and ProGlide (*n* = 819) techniques after TAVI (Table 1). Both RCTs included overall 360 patients treated with the plug-based technique and 362 patients treated with the suture-based technique. The main study characteristics are summarized in Table 1. Major baseline characteristics of all included studies are presented in Table 2.

**Table 1:** Characteristics of the included studies

Study	Study type	Multicenter study	Patients with MANTA/ProGlide VCD	Used size of MANTA VCD	One or two ProGlide VCDs	Methods for vascular access	Procedure	Anti-coagulation during procedure	Anti-coagulation reversal	Years of enrollment	Follow up time
Choice-closure [15]	RCT	Yes (3 centers)	258/258	18 French	2 ProGlides	Angiography or ultrasound guided	TAVI	UFH	Protamine	06/2020–06/2021	30 days
MASH [14]	RCT	Yes (2 centers)	102/104	N/A	2 ProGlides	Ultrasound guided	TAVI	UFH	Protamine	10/2018–01/2020	< 30 days
Ali et al. [13]	OS	No	50/86	N/A	2 ProGlides	Angiography or ultrasound guided	TAVI	UFH	Protamine	11/2017–06/2018	30 days
Biancari et al. [8]	OS	No	107/115	14 and 18 French	2 ProGlides	Angiography or ultrasound guided	TAVI	N/A	N/A	09/2016–10/2017	30 days
Dumpies et al. [9]	OS	No	195/383	18 French	Both	Angiography or ultrasound guided	TAVI	UFH	Protamine	02/2019–09/2019	Hospital discharge
Medranda. et al. [10]	OS (Propensity matched)	No	124/124	14 and 18 French	2 ProGlides	Ultrasound guided	TAVI	UFH	Protamine	08/2017–08/2020	N/A
Moriyama et al. [11]	OS (Propensity matched)	No	111/111	18 French	2 ProGlides	N/A	TAVI	UFH	Protamine	01/2016–03/2018	Hospital discharge

*ACT* activated clotting time; *N/A* not available; *sec* seconds; *OS* observational study; *RCT* randomized controlled trial; *TAVI* transcatheter aortic valve implantation; *UFH* unfractionated heparin; *VCD* vascular closure device

**Table 2:** Baseline characteristics

Groups	Randomized controlled trials				Observational studies									
	CHOICE-CLOSURE [15]		MASH [14]		Ali et al. [13]		Biancari et al. [8]		Dumpies et al. [9]		Medranda et al. [10]		Moriyama et al. [11]	
	MANTA	ProGlide	MANTA	ProGlide	MANTA	ProGlide	MANTA	ProGlide	MANTA	ProGlide	MANTA	ProGlide	MANTA	ProGlide
Patients	*n* = 258	*n* = 258	*n* = 102	*n* = 104	*n* = 50	*n* = 86	*n* = 107	*n* = 115	*n* = 195	*n* = 383	*n* = 124	*n* = 124	*n* = 111	*n* = 111
Age (years)	80.7 ± 5.7	80.4 ± 6.5	81 (76–85)	82 (74–84)	81.5 ± 8.0	79.1 ± 6.8	79.8 ± 6.0	80.7 ± 6.8	80.9 ± 6.3	80.1 ± 6.1	77.5 ± 8.7	76.9 ± 9.4	79.5 ± 7.1	79.8 ± 7.2
Male	55.4%	55.4%	52.0%	55.8%	57.0%	56.0%	48.3%	45.2%	55.4%	47.8%	63.7%	74.2%	43.2%	41.4%
BMI in kg/m^2^	28.5 ± 5.1	28.6 ± 5.3	26 (24–29)	26 (23–29)	28.5 ± 6.0	28.2 ± 4.6	27.3 ± 4.8	28.0 ± 5.0	29.2 ± 6.1	28.8 ± 11.8	N/A	N/A	26.7 ± 4.8	27.6 ± 5.5
Hypertension	89.5%	92.6%	73.5%	68.3%	N/A	N/A	N/A	N/A	89.2%	90.3%	87.8%	87.3%	85.6%	91.0%
Diabetes	38.0%	39.9%	23.5%	22.1%	24.0%	30.2%	25.2%	26.1%	42.6%	42.6%	42.2%	35.0%	24.3%	21.6%
eGFR (ml/min)	58.0 (42.0–73.3)	57.0 (41.0–70.0)	60 (46–78)	63 (47–80)	N/A	N/A	N/A	N/A	54.7 ± 20.3	54.7 ± 20.0	N/A	N/A	66.8 ± 23.3	63.5 ± 21.2
Dialysis	N/A	N/A	N/A	N/A	N/A	N/A	2.8%	0.9%	3.1%	3.4%	3.2%	4.0%	N/A	N/A
PAD	7.0%	8.1%	5.8%	2.9%	4.7%	2.0%	9.3%	9.6%	10.8%	9.4%	10.1%	11.3%	18.0%	16.2%
CAD	54.7%	45.7%	N/A	N/A	N/A	N/A	56.1%	53.0%	58.5%	59.8%	N/A	N/A	N/A	N/A
Previous PCI	24.0%	23.6%	36.3%	30.8%	8.0%	19.6%	27.1%	21.7%	20.5%	30.3%	32.4%	21.5%	21.6%	21.6%
Previous CABG	9.7%	8.1%	5.9%	9.6%	N/A	N/A	N/A	N/A	12.3%	6.5%	17.3%	17.4%	4.5%	9.0%
Pre-procedure oral anticoagulation	32.3%	42.7%	30.4%	27.9%	30.0%	31.4%	38.3%	39.1%	45.6%	43.9%	N/A	N/A	38.7%	37.8%
STS Score in (%)	4.5 ± 4.8	4.6 ± 4.3	2.7 (1.8–4.3)	2.8 (1.6–3.9)	N/A	N/A	N/A	N/A	5.1 ± 3.4	4.4 ± 3.4	3.4 ± 2.9	5.0 ± 3.7	4.3 ± 2.9	4.3 ± 3.2
EuroScore II in (%)	4.5 ± 4.8	4.6 ± 4.3	2.6 (1.9 -3.6)	2.4 (1.6–4.3)	N/A	N/A	4.4 ± 3.7	4.4 ± 3.2	N/A	N/A	N/A	N/A	4.4 ± 3.3	4.6 ± 3.9
Moderate to severe access calcification	49.2%	48.8%	37.3%	41.3%	22.0%	24.7%	N/A	N/A	22.1%	19.8%	36.3%	44.5%	N/A	N/A
Self-expanding valves	60.5%	62.8%	32.4%	33.7%	N/A	N/A	55.1%	27.8%	55.9%	66.1%	N/A	N/A	52.3%	22.5%
Balloon-expandable valves	34.5%	33.7%	62.7%	52.9%	44.4%	45.2%	44.9%	72.2%	34.4%	32.9%	N/A	N/A	47.7%	51.4%
Mechanically expanding valves	5.0%	3.5%	3.9%	13.5%	N/A	N/A	0%	0%	9.2%	1.0%	N/A	N/A	0%	26.1%

Values are mean ± SD or median (interquartile range) or %

*BMI* body mass index; *CAD* coronary artery disease; *eGFR* estimated glomerular filtration rate; *N/A* not available; *PAD* peripheral artery disease; *PCI* percutaneous coronary intervention; *STS* Society of Thoracic Surgeons

### Observational studies

**Fig. 2:**
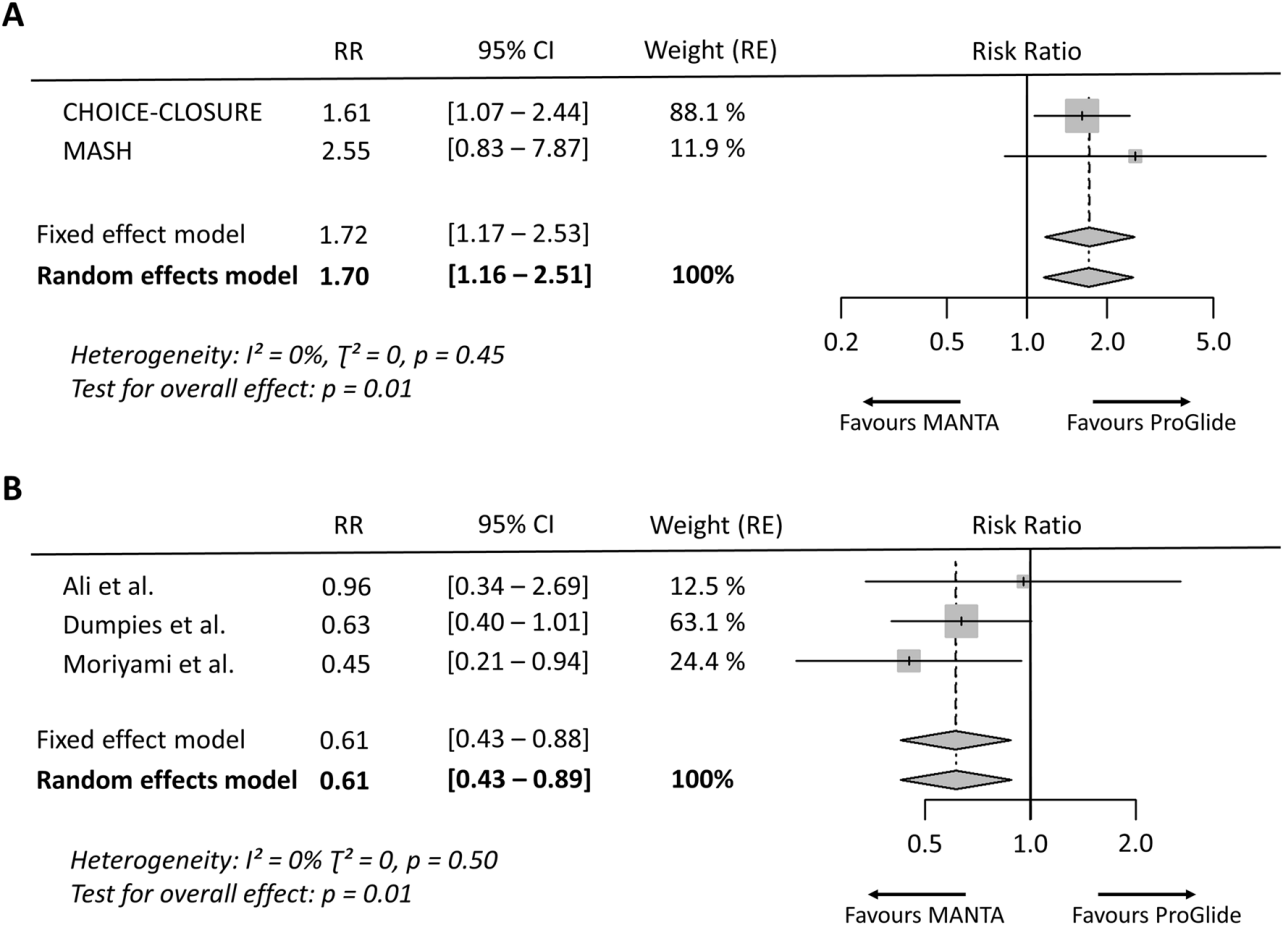
Access-site related vascular complications after MANTA versus ProGlide vascular closure. **A** Access-site related vascular complications from randomized controlled trials. **B** Access-site related vascular complications from observational studies. Size of data markers indicates weight of study in the pooled analysis. *RE* random effects model; *RR* risk ratio

**Fig. 3:**
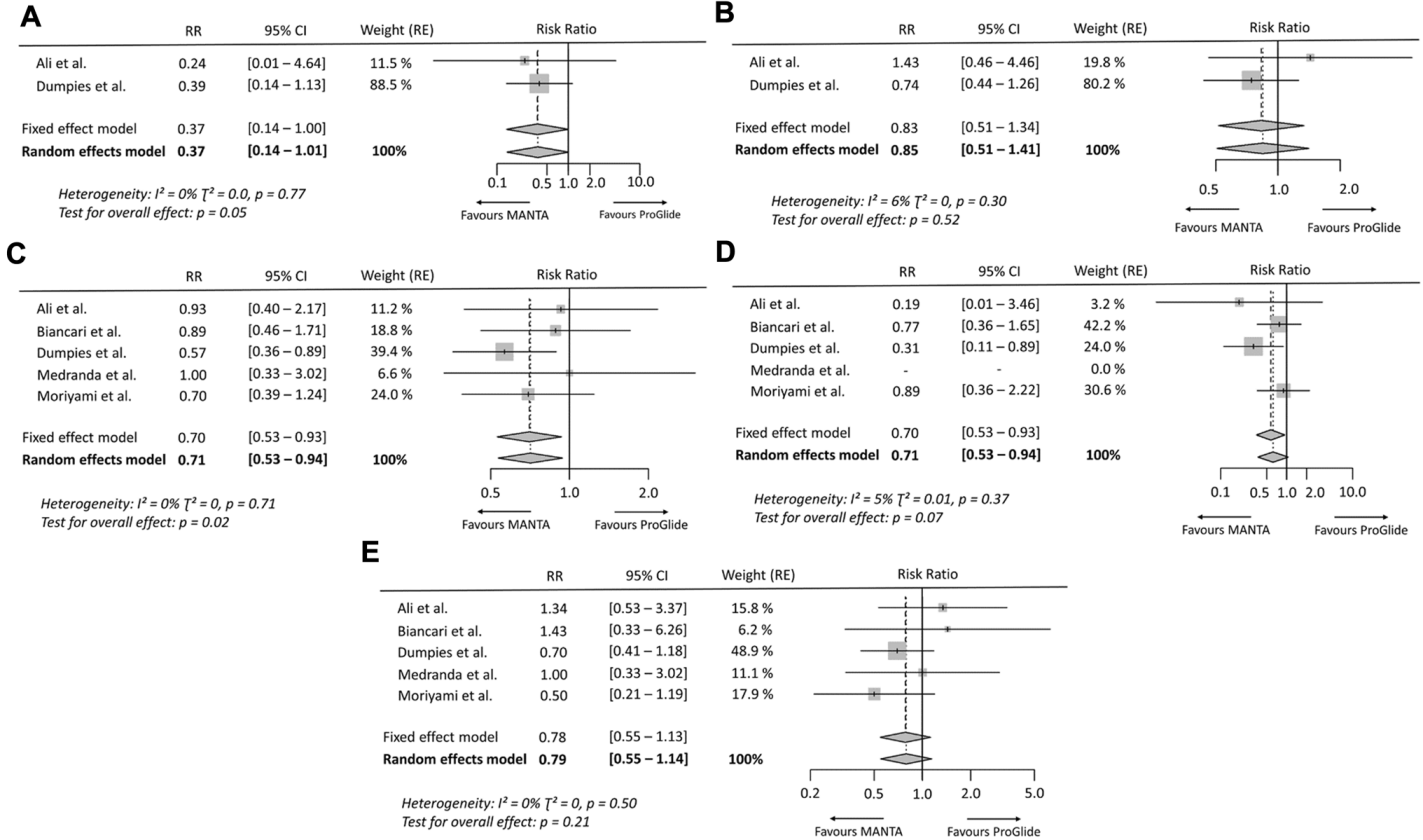
Risk ratio for vascular complications of observational studies. **A** Major access-site related vascular complications, **B** minor access-site related vascular complications, **C** overall vascular complications, **D** major vascular complications, and **E** minor vascular complications. Size of data markers indicates weight of study in the pooled analysis. *RE* random effects model; *RR* risk ratio

In the OS group, overall access-site related vascular complications (RR 0.61 [95% CI 0.43–0.89], *p* = 0.01, *I*^2^ = 0%) (Fig. 2) and overall vascular complications (RR 0.71 [95% CI 0.53–0.94], *p* = 0.02, *I*^2^ = 0%) were significantly less frequent after application of the MANTA technique. The individual events access-site related major (RR 0.37 [95% CI 0.14–1.01], *p* = 0.05, *I*^2^ = 0%) and access-site related minor vascular complications (RR 0.85 [95% CI 0.51–1.41], *p* = 0.52, *I*^2^ = 6%) did not differ significantly, as well as overall major (RR 0.62 [95% CI 0.37–1.05], *p* = 0.07, *I*^2^ = 5%) and minor vascular complications (RR 0.79 [95% CI 0.55–1.14], *p* = 0.21, *I*^2^ = 0%) (Fig. 3). There was also no relevant difference in the rate of VCD failure (RR 0.82 [95% CI 0.49–1.38], *p* = 0.46, *I*^2^ = 0%) and endovascular stenting or surgery due to device failure (RR 1.13 [95% CI 0.53–2.41], *p* = 0.76, *I*^2^ = 0%) between both techniques. The analysis of overall bleeding events (RR 0.57 [95% CI 0.32–1.02], *p* = 0.06 *I*^2^ = 70%) and the subtypes of life-threatening or major bleeding (RR 0.39 [95% CI 0.11–1.37], *p* = 0.14, *I*^2^ = 72%) and minor bleeding (RR 0.59 [95% CI 0.33–1.06], *p* = 0.08, *I*^2^ = 0.0%) showed a trend but no significant difference between the plug-based and the suture-based techniques. There was also no significant difference in mortality between both VCD strategies (RR 0.51 [95% CI 0.17–1.51], *p* = 0.22, *I*^2^ = 0%) (supplementary Fig. 1).

### Randomized controlled trials

**Fig. 4:**
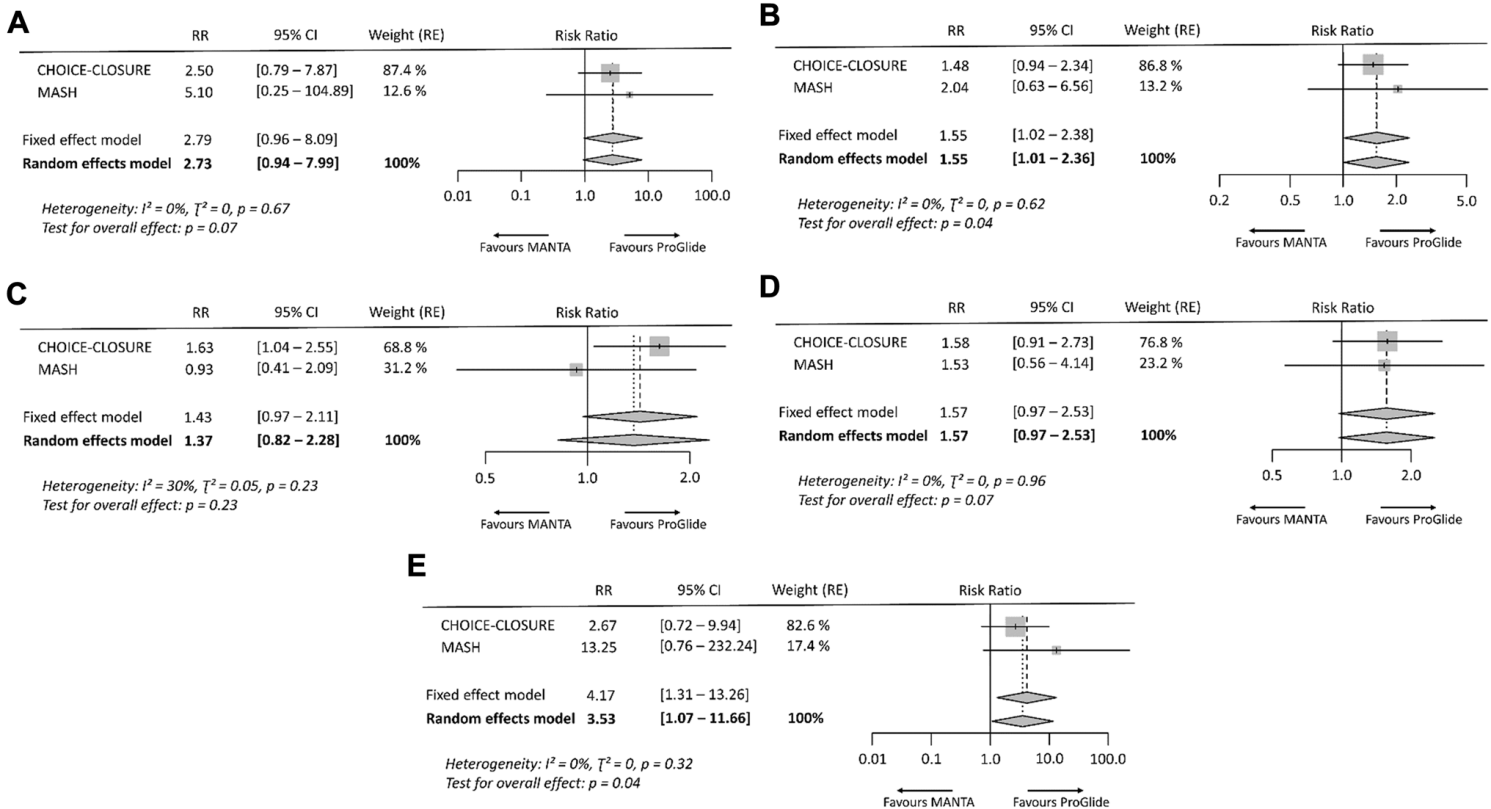
Risk ratio for vascular events, bleeding events and endovascular stenting or vascular surgery due to vascular closure device failure of randomized controlled trials. **A** Major access-site related vascular complications, **B** minor access-site related vascular complications, **C** overall bleeding events, **D** access-site related bleeding events, **E** stenting or vascular surgery due to vascular closure device failure. Size of data markers indicates weight of study in the pooled analysis. *RE* random effects model; *RR* risk ratio

In the analysis of RCT data, overall access-site related vascular complications occurred significantly more frequently after vascular closure with the MANTA technique (RR 1.70 [95% CI 1.16–2.51], *p* = 0.01, *I*^2^ = 0%) (Fig. 2). Similarly, the rate of minor access-site related vascular complications was significantly higher in the MANTA cohort (RR 1.55 [95% CI 1.01–2.36], *p* = 0.04, *I*^2^ = 0%), but not that of major access-site related vascular complications (RR 2.73 [95% CI 0.94–7.99], *p* = 0.07, *I*^2^ = 0%). Endovascular stenting or vascular surgery due to VCD failure had to be performed more frequently after MANTA VCD application (RR 3.53 [95% CI 1.07–11.66], *p* = 0.04, *I*^2^ = 0%). No significant difference was observed between the two techniques in terms of overall bleeding (RR 1.37 [95% CI 0.82–2.28], *p* = 0.23, *I*^2^ = 30%) and access-site related bleeding (RR 1.57 [95% CI 0.97–2.53], *p* = 0.07, *I*^2^ = 0%) (Fig. 4).

## Discussion

The main findings of this study are as follows: (1) Overall access-site related vascular complications appear to be more common in the MANTA cohort of the RCTs; (2) patients in the MANTA cohort of RCTs were more likely to undergo vascular stenting or vascular surgery due to VCD failure; (3) in contrast to the RCT data, the results of the OS meta-analysis show better outcomes with the MANTA VCD in terms of overall access-site related vascular complications.

In contrast to our analysis, none of the previously published meta-analyses could find a difference between the two VCDs in terms of vascular complications [16–18]. Most likely, this is due to mixing of RCTs and OS in previous meta-analyses, possibly leading to a high risk of bias and heterogeneity of results. Moreover, given the nearly diametric results in both types of studies, a joint analysis seems even more questionable. Furthermore, all previous meta-analyses did not differentiate between access-site related vascular complications and overall vascular complications (including non-access-site related ones). Furthermore, two of the previous meta-analyses included the ProStar device in the cohort of suture based VCD [16, 18], and previous studies have already demonstrated that the ProStar device is inferior to ProGlide [1].

Different event rates were observed in both RCTs and OS, particularly with regard to vascular complications. Presumably, these differences are a consequence of different assessment, adjudication and follow-up strategies. An important example is the structured ultrasound follow-up in the CHOICE-CLOSURE trial. Compared with clinical follow-up alone, routine ultrasound may be able to diagnose vascular complications more accurately, particularly minor ones.

Plug-based vascular closure is a commonly applied technique for small-sized arterial access and is associated with favorable outcome [24, 25]. The fact that these outcomes cannot be reproduced with large-bore vascular access using the MANTA VCD may have several reasons. First, operators have a higher cumulative experience with the application of suture-based closure techniques in the setting of procedures requiring large-bore vascular access. However, it should be mentioned that the MANTA VCD has a short learning curve [11, 26], and the principal concepts of plug-based techniques should be familiar to the majority of operators. Second, the MANTA VCD seems to be more prone to misplacement resulting in vascular complications than the suture-based ProGlide VCD. Mocetti et al. previously described several failure mechanisms that could lead to device failure and pseudoaneurysms [26]. In addition, the MANTA VCD appears to achieve faster hemostasis, which may provide a false sense of security and lead to unnoticed bleeding, which may potentially explain the significantly higher incidence of pseudoaneurysms after MANTA application in the CHOICE-CLOSURE trial [15]. Moriyama et al. demonstrated that with the help of ultrasound guidance, device failure could be mitigated, with lower rate of access-site related vascular and bleeding complications [27]. Furthermore, van Wiechen et al. found a small vessel diameter and a high or low puncture height in relation to the femoral bifurcation to be associated with a poor outcome of the MANTA VCD [28]. In addition, Kmiec et al. described female sex, vascular access-site angulation and more than mild vascular calcification of the dorsal vessel segment as other potential predictors of MANTA device failure and vascular complications [29]. Therefore, optimizing patient selection and application of the device could possibly improve outcomes. The disparate results of OS and RCTs could also suggest that an unselected use of the MANTA VCD yields worse outcomes. Nevertheless, the higher cost of the MANTA device must be balanced against its potential advantages in everyday clinical practice, especially since the MASH [14] and CHOICE-CLOSURE studies [15] found no advantage in unselected use of the MANTA VCD over the ProGlide technique.

Another procedural advantage of the ProGlide technique is the fact that wire access to the vessel is maintained during vascular closure. This enables the operator to apply additional VCDs even if the device does not achieve full hemostasis. In contrast, once the plug-based VCD fails, there are only a few bail-out options such as endovascular stenting or vascular surgery. Nevertheless, the MANTA VCD has already been successfully used in several cases as a bail-out device after ProGlide failure [14, 15, 30], which could possibly reduce the need for endovascular stenting or vascular surgery after suture-based VCD failure. A detailed investigation of the MANTA device as a bail-out option is currently missing.

Plug-based VCDs could represent an additional option for other procedures requiring large-bore vascular closure such as thoracic endovascular aortic repair (TEVAR), EVAR and va-ECMO. Initial registry studies and case reports have already shown that plug-based VCDs could be a safe alternative to the ProGlide VCD [31–33]. However, comparative and randomized studies are still lacking in this setting and could therefore not be included in this meta-analysis.

The results of our study reinforce the importance of adequately powered RCTs for the evaluation of new procedures and devices. Even OS with a large number of included patients (Dumpies et al. [9] and Medranda et al. [10]) or methodologically sophisticated propensity-matched analyses (Medranda et al. [10] and Moriyama et al. [11]), could not anticipate the results obtained by the RCTs, both of which pointed in the same direction leading to a low grade of statistical heterogeneity. Accordingly, the meta-analysis of OS could not find any different results other than those previously published, as there seems to be a relevant bias in all non-randomized comparisons between the MANTA and ProGlide VCD.

There are several confounders that may have influenced the outcome of OS. First, a certain selection bias cannot be excluded in studies that used both VCDs in parallel (Ali et al. [13], Biancari et al. [8], Dumpies et al. [9] and Medranda et al. [10]). For the OS that introduced the MANTA device after the ProGlide device, it can be suspected that the operators’ growing experience with large-bore access may have positively influenced the results of the plug-based VCD (Moriyama et al. [11]). Propensity-matched analyses can create virtual equality between the two VCD groups with respect to measured confounding factors and baseline characteristics. Nevertheless, some important variables, such as the exact access-site vascular characteristics or puncture details, are too complex to be accurately included in such an analysis and possible unknown factors cannot be accounted for. Finally, RCTs remain the reference standard for comparing treatment options with a low risk of bias due to unmeasured confounders.

### Limitations

Our meta-analysis has several limitations. First, this analysis is mainly based on published data. We did not have access to data on the patient level, except for CHOICE-CLOSURE [15] and Dumpies et al. [9]. Second, it was not possible to collect all outcome data targeted in the planning phase of the meta-analysis from all included studies (supplementary table 3). Third, only two randomized trials were published and therefore available for inclusion in this meta-analysis. Fourth, all included comparative studies analyzed only large-bore vascular closure after TAVI. Therefore, no statement can be made regarding the use of the MANTA VCD after TEVAR, EVAR or va-ECMO. Finally, all included OS show a relevant risk of bias in comparing the MANTA and ProGlide VCD (supplementary table 2).

## Conclusion

RCT data show that large-bore vascular closure with the MANTA-based technique is associated with a significantly increased rate of access-site related vascular complications as well as endovascular stenting or vascular surgery due to device failure compared with the ProGlide-based technique. The meta-analysis of OS provided opposite findings. These results highlight the importance of high-quality RCTs as evidence for guiding treatment decisions.

## Supplementary Information

Below is the link to the electronic supplementary material.

Supplementary file1 (DOCX 131 KB)

## Data Availability

Upon request.

## Abbreviations

EVAR: Endovascular aortic repair
OS: Observational studies
RCT: Randomized controlled trial
RR: Risk ratio
TAVI: Transcatheter aortic valve implantation
va-ECMO: Venoarterial extracorporeal membrane oxygenation
VARC: Valve academic research consortium
VCD: Vascular closure device

## Code availability

R (version 4.1.2) and its package meta (version 5.1-1) for all statistical analyses.
