# Supplementary material for: Comparison of plug-based versus suture-based vascular closure for large-bore arterial access: a collaborative meta-analysis of observational and randomized studies
Source: Clin Res Cardiol. 2023 Feb 7;112(5):614–25. doi: 10.1007/s00392-022-02145-5 (PMC10160216; doi:10.1007/s00392-022-02145-5)
Supplement: Supplementary file 1 — Supplementary file1 (DOCX 131 KB) [file 392_2022_2145_MOESM1_ESM.docx]

**SUPPLEMENTARY MATERIAL**

**SUPPLEMENTARY TABLES**

**Supplementary table 1: Trial quality and risk of bias of randomised trials according to RoB-2 (Risk of Bias 2 tool)**

| **Trials (Ref. #)** | **Randomisation process** | **Deviation from intended interventions** | **Missing outcome data** | **Measurement of outcome** | **Selection of reported results** | **Overall** |
| --- | --- | --- | --- | --- | --- | --- |
| **CHOICE-CLOSURE (1)** | low | high | low | low | low | Some concerns |
| **MASH (2)** | low | high | some concerns | some concerns | low | Some concerns |

**Supplementary table 2: Trial quality and risk of bias of non-randomised trials according to ROBINS-I (Risk Of Bias In Non-randomised Studies of Interventions)**

| **First author (Ref. #)** | Bias due to confounding | Bias in selection of participants | Bias in classification of interventions | Bias due to departures from intended interventions | Bias due to missing data | Bias due to measurement of outcomes | Bias in selection of reported results | Overall risk of bias judgment |
| --- | --- | --- | --- | --- | --- | --- | --- | --- |
| Ali et al. (3) | Serious | Low | Low | Low | Low | Moderate | Low | Serious |
| Biancari et al. (4) | Serious | Low | Low | Low | Low | Moderate | Low | Serious |
| Dumpies et al. (5) | Serious | Low | Low | Low | Low | Moderate | Low | Serious |
| Medranda. et al. (6) | Moderate | Low | Low | Low | No informations | Moderate | Low | Moderate |
| Moriyama et al. (7) | Moderate | Low | Low | Low | No informations | Moderate | Low | Moderate |

**Supplementary table 3: Trial definitions and outcome assessment**

| **Trials (Ref.#)** | **Vascular complications**  **- definition** | **Vascular complications**  **-assessment** | **Bleeding events**  **- definition** | **Bleeding events**  **- assessment** | **Vascular closure device failure**  **- definition** | **Overall mortality**  **- assessment** |
| --- | --- | --- | --- | --- | --- | --- |
| **CHOICE-CLOSURE (1)** | VARC-2 | overall (major/minor) and access site related vascular complications  (major/minor) | VARC-2 | overall (life threatening/ major/minor) and access site related bleeding (life threatening /major/minor) | VARC-2 | in-hospital and after 30 days |
| **MASH**  **(2)** | VARC-2 | access site related vascular complications  (major/minor) | VARC-2 | overall (any) and access site related bleeding (any) | Failure of the vascular closure device to achieve  hemostasis within 5 min or requiring additional  endovascular maneuvers (endovascular  stenting, surgical techniques, or additional closure  devices) | not assessed |
| **Ali et al.**  **(3)** | VARC-2 | overall (major/minor) and access site related vascular complications  (major/minor) | VARC-2 | overall bleeding  (life threatening or major/minor) and access site related bleeding (life threatening or major/minor) | VARC-2 | after 30 days |
| **Biancari et al.**  **(4)** | VARC-2 | overall vascular complications  (major/minor) | VARC-2 | Overall bleeding  (life threatening/major) | VARC-2 | in-hospital and after 30 days |
| **Dumpies et al.**  **(5)** | VARC-2 | overall (major/minor) and access site related vascular complications  (major/minor) | VARC-2 | Overall bleeding  (life threatening/ major/minor) | VARC-2 | in-hospital |
| **Medranda. et al.**  **(6)** | VARC-2 | overall vascular complications  (major/minor) | not mentioned | Access site haematoma | VARC-2 | in-hospital |
| **Moriyama et al.**  **(7)** | VARC-2 | overall (major/minor) and access site related vascular complications (any) | VARC-2 | Overall bleeding  (life threatening/ major/minor) | VARC-2 | in-hospital |
| VARC-2: Valve Academic Research Consortium-2 | | | | | | |

**Supplementary table 4: Trial inclusion and exclusion criteria**

| **Trials (Ref. #)** | **Major inclusion criteria** | **Exclusion criteria** |
| --- | --- | --- |
| **CHOICE-CLOSURE**  **(1)** | - judged by the local heart team for TAVI - transfemoral access route - commercially-available transcatheter aortic valve | - vascular access site anatomy not suitable for percutaneous vascular closure - the occurrence of vascular access site complications prior to the TAVI procedure - known allergy or hypersensitivity to any VCD component - unstable active bleeding or bleeding diathesis or significant unmanageable anemia - absence of computed tomographic data of the access site before the procedure - systemic infection or a local infection at or near the access site - life expectancy of less than 6 months due to non-cardiac conditions - patients that cannot adhere to or complete the investigational protocol for any reason - pregnant or nursing subjects - participation in any other interventional trial. |
| **MASH**  **(2)** | - Patients undergoing elective transfemoral TAVI for severe aortic valve stenosis with any commercially-available transcatheter heart valve (THV) - Common femoral artery diameter > 5.0mm (14 - 22F compatible) | - Symptomatic leg ischaemia - Previous thromboendarterectomy or plastic patch of the common femoral artery - Previous implantation of a suture-based VCD less than 30 days before, or a plug-based VCD within 6 months - Unilateral or bilateral lower extremity amputation - Systemic infection or a local infection at or near the access site - Allergy to the components any of both devices (i.e. bovine materials or any other device material, including collagen and/or collagen products, polyglycolic or polylactic acid, stainless steel or nickel) - Active bleeding or bleeding diathesis including thrombocytopenia (platelet count <50,000 cells/UL), thrombasthenia, hemophilia, or von Willebrand disease - Patients in whom continuous oral anticoagulation therapy cannot be stopped for the peri-procedural period or patients with INR >1.8 at the time of the procedure - Patient unable to be adequately anti-coagulated for the procedure - Morbidly obese or cachectic (BMI >40 kg/m2 or <20 kg/m2) - Anatomical and procedural contraindication for suture-based or Manta closure - Lack of proper puncture site in the common femoral artery in terms of calcification, size, and atherosclerotic disease - Absence of computed tomographic data of the access site before the procedure - Patient cannot adhere to or complete the investigational protocol for any reason including but not limited to geographical residence, psychiatric condition or life threatening disease - Known pregnancy at time of randomization (in women of childbearing potential a negative pregnancy test is mandatory) - Participating in trials in which the primary endpoint includes bleeding or vascular complications |
| **Ali et al.**  **(3)** | - TAVI - transfemoral access route | - non-transfemoral TAVI - planned femoral surgical cut-down |
| **Biancari et al.**  **(4)** | - TAVI - transfemoral access route | - not mentioned |
| **Dumpies et al.**  **(5)** | - TAVI - transfemoral access route | - non-transfemoral TAVI - surgical cut-down |
| **Medranda. et al.**  **(6)** | - TAVI - transfemoral access route - Propensity matched | - non-transfemoral TAVI |
| **Moriyama et al.**  **(7)** | - TAVI - transfemoral access route - Propensity matched | - non-transfemoral TAVI - surgical cut-down - death during procedure - conversion to open heart surgery |
| TAVI: transfemoral aortic valve implantation; VCD: vascular closure device | | |

**SUPPLEMENTARY FIGURES**

**
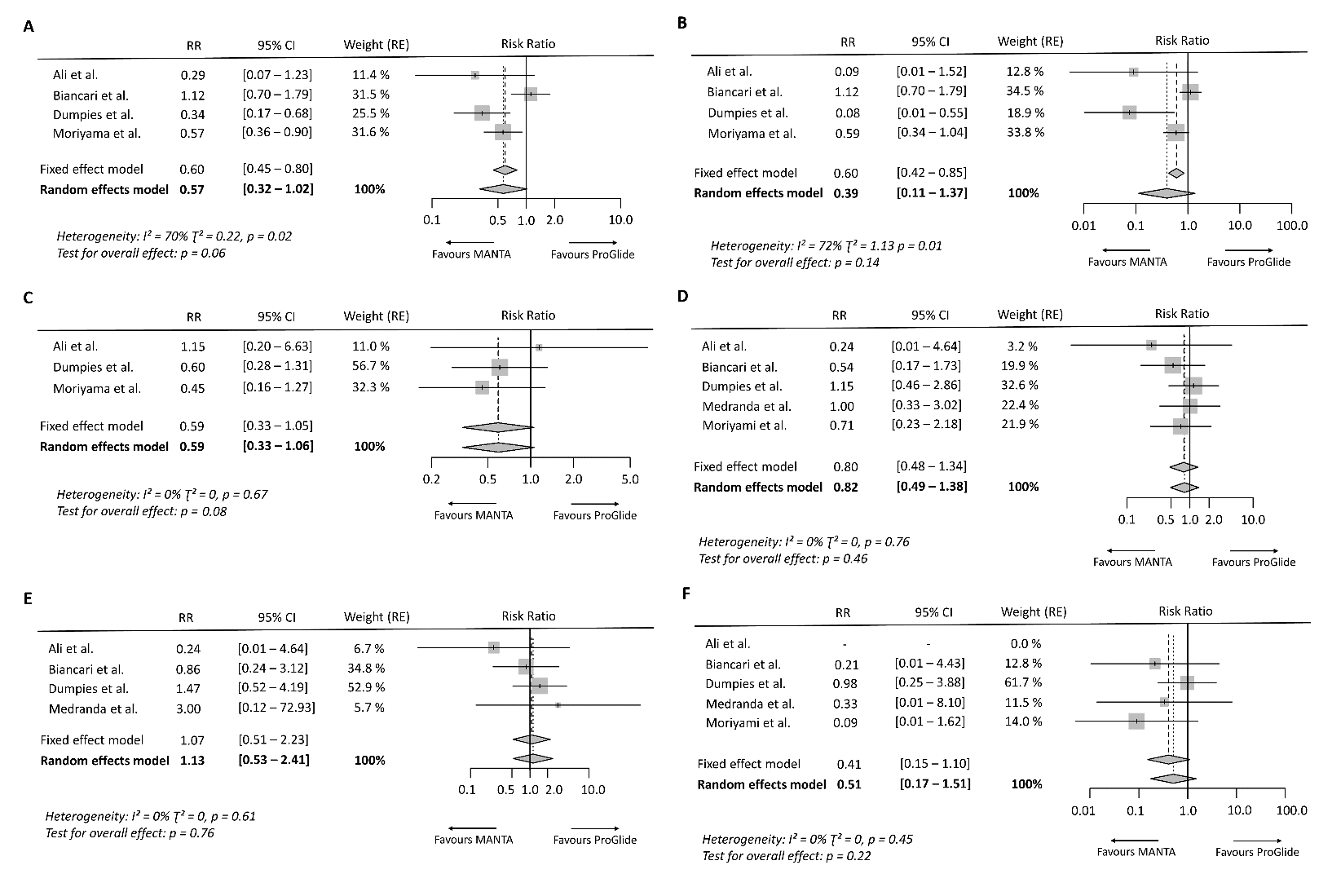
**

**Supplementary figure 1: Risk ratio for bleeding complications, device failure and mortality of observational studies** A) Overall bleeding events B) Life-threatening or major bleeding events C) Minor bleeding events D) Vascular closure device failure E) Endovascular stenting or surgery due to vascular closure device failure F) Mortality. Size of data markers indicates weight of study in the pooled analysis. RE: Random effects model. RR: Risk Ratio.

**References**

1. Abdel-Wahab M, Hartung P, Dumpies O, Obradovic D, Wilde J, Majunke N, Boekstegers P, Müller R, Seyfarth M, Vorpahl M, Kiefer P, Noack T, Leontyev S, Sandri M, Rotta Detto Loria J, Kitamura M, Borger MA, Funkat A-K, Hohenstein S, Desch S, Holzhey D, Thiele H. Comparison of a Pure Plug-Based versus a Primary Suture-Based Vascular Closure Device Strategy for Transfemoral Transcatheter Aortic Valve Replacement: The CHOICE-CLOSURE Randomized Clinical Trial. *Circulation*.2021.

2. van Wiechen MP, Tchétché D, Ooms JF, Hokken TW, Kroon H, Ziviello F, Ghattas A, Siddiqui S, Laperche C, Spitzer E, Daemen J, Jaegere PP de, Dumonteil N, van Mieghem NM. Suture- or Plug-Based Large-Bore Arteriotomy Closure: A Pilot Randomized Controlled Trial. *JACC Cardiovasc Interv*.2021;14:149–57.

3. Ali N, Dospinescu C, Cunnington M, Malkin C, Blackman D. A Comparison of Efficacy, Safety and Cost Between MANTA TM and Proglide Vascular Closure Devices Following Transfemoral Transcatheter Aortic Valve Implantation. *Heart Research - Open Journal*.2021.

4. Biancari F, Romppanen H, Savontaus M, Siljander A, Mäkikallio T, Piira O-P, Piuhola J, Vilkki V, Ylitalo A, Vasankari T, Airaksinen JKE, Niemelä M. MANTA versus ProGlide vascular closure devices in transfemoral transcatheter aortic valve implantation. *Int J Cardiol*.2018;263:29–31.

5. Dumpies O, Kitamura M, Majunke N, Hartung P, Haag A, Wilde J, Desch S, Sandri M, Crusius L, Noack T, Kiefer P, Leontyev S, Borger M, Thiele H, Holzhey D, Abdel-Wahab M. Manta versus Perclose ProGlide vascular closure device after transcatheter aortic valve implantation: Initial experience from a large European center. *Cardiovasc Revasc Med*.2021.

6. Medranda GA, Case BC, Zhang C, Rappaport H, Weissman G, Bernardo NL, Satler LF, Ben-Dor I, Rogers T, Waksman R. Propensity-matched comparison of large-bore access closure in transcatheter aortic valve replacement using MANTA versus Perclose: A real-world experience. *Catheter Cardiovasc Interv*.2021;98:580–5.

7. Moriyama N, Lindström L, Laine M. Propensity-matched comparison of vascular closure devices after transcatheter aortic valve replacement using MANTA versus ProGlide. *EuroIntervention*.2019;14:e1558-e1565.
